# Effective Multi-Object Tracking via Global Object Models and Object Constraint Learning

**DOI:** 10.3390/s22207943

**Published:** 2022-10-18

**Authors:** Yong-Sang Yoo, Seong-Ho Lee, Seung-Hwan Bae

**Affiliations:** Vision and Learning Laboratory, Department of Computer Engineering, Inha University, Incheon 22212, Korea

**Keywords:** multi-object tracking, global appearance model, global relation motion model, object constraint learning, affinity model, surveillance system

## Abstract

Effective multi-object tracking is still challenging due to the trade-off between tracking accuracy and speed. Because the recent multi-object tracking (MOT) methods leverage object appearance and motion models so as to associate detections between consecutive frames, the key for effective multi-object tracking is to reduce the computational complexity of learning both models. To this end, this work proposes global appearance and motion models to discriminate multiple objects instead of learning local object-specific models. In concrete detail, it learns a global appearance model using contrastive learning between object appearances. In addition, we learn a global relation motion model using relative motion learning between objects. Moreover, this paper proposes object constraint learning for improving tracking efficiency. This study considers the discriminability of the models as a constraint, and learns both models when inconsistency with the constraint occurs. Therefore, object constraint learning differs from the conventional online learning for multi-object tracking which updates learnable parameters per frame. This work incorporates global models and object constraint learning into the confidence-based association method, and compare our tracker with the state-of-the-art methods on public available MOT Challenge datasets. As a result, we achieve 64.5% MOTA (multi-object tracking accuracy) and 6.54 Hz tracking speed on the MOT16 test dataset. The comparison results show that our methods can contribute to improve tracking accuracy and tracking speed together.

## 1. Introduction

Multi-object tracking (MOT) is the problem of finding states of multiple objects, and then associating them accurately between consecutive frames. MOT has been applied to many applications, such as surveillance systems, autonomous driving, and sport analysis over recent years. The tracking-by-detection paradigm is usually employed to solve the MOT problem, and it has achieved impressive performance improvements. Once detection responses are provided by a detector at each frame, the detections are linked (*or* associated) across the frames.

According to the association manners, the tracking-by-detection methods can be categorized into batch and online methods. Batch-based MOT methods [1,2,3,4,5,6,7,8] exploit the detection results of all frames. They can build longer tracks under occlusions and with incomplete detections since they can achieve (temporal) global associations between long frames. However, they cannot be utilized for the real-time system since they exploit detections of all frames for iterative global association. On the other hand, online MOT methods [9,10,11,12,13,14,15,16,17] sequentially build tracks based on the (temporal) local association with up to current frame detections. For that reason, online MOT methods can suffer from occluded objects or false detection rather than batch methods.

For achieving high-quality MOT, improving data association is still important, and it can be attained, usually, by improving affinity models for tracked objects. Object appearance and motion models are used frequently since they are important cues for differentiating objects. For improving the discriminability, object-specific model learning has flourished, which generates and updates each object model independently. Refs. [14,15,18] apply single object tracking (SOT) methods into MOT for generating a local object feature. In particular, Ref. [13] exploits a SOT sub-network to capture short-term cues, and uses them for modeling local interactions between objects and discriminating objects.

In [19], two ResNet-50 [20] networks are utilized for more accurate association, respectively. However, the computational complexity of learning each object model is a significant burden since the complexity is proportional to the number of objects.

For more effective association, several global object models have been developed [9,21,22,23]. Ref. [24] evaluates the affinity by using a sub-network to fuse discriminative appearance and motion information. Ref. [25] utilizes a CNN-based correlation filter for tracking multiple objects with geometric and appearance features.

Even though leveraging global object models for MOT is beneficial for reducing the object model complexity, it is still challenging to develop an effective MOT method due to the trade-off between tracking accuracy and speed. For effective MOT, we, therefore, propose global object models which can discriminate different tracked objects accurately while keeping low training and inference complexity. For our global models, we first design a global appearance model using contrastive learning. Specifically, we extract high-level semantic object features for tracked objects from a light ConvNet. We then define positive (i.e., sample) and negative (i.e., different) object feature pairs in consideration of their identifications. Based on the triplet loss [26], we then minimize the feature distance between positive pairs, whereas maximize that of negative object pairs.

**Figure 1:**
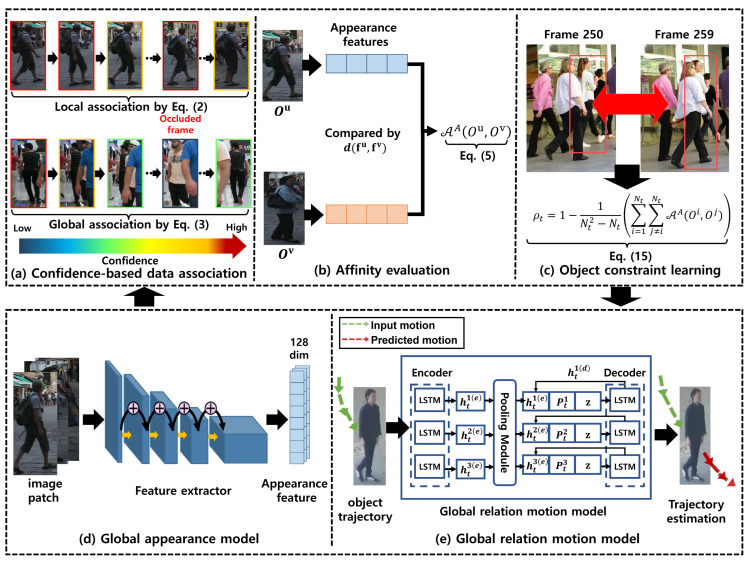
The proposed MOT framework based on our global models and object constraint learning algorithm. We describe our multi-object tracker in the upper box, and global models in the bottom box. Our multi-object tracker consists of three parts. In (**a**), we describe the confidence-based data association. During multi-object tracking, we calculate the affinity scores as depicted in (**b**). To determine a frame of updating our global appearance model, we exploit the object constraint learning algorithm as depicted in (**c**). We use our global appearance and motion models to improve the data association quality. Our global appearance and motion models are shown in (**d**,**e**), respectively.

In addition, we present a global relation motion model to predict object future trajectories from their previous motions. We use each object past trajectory, and relative motions between tracked objects as the main motion cues of the global relation motion model. To improve motion prediction results, our global relation motion model mainly consists of a generator and a discriminator, which are trained by using adversarial learning [27]. Even though our global appearance and motion models are effective for computation, online learning these models at each frame increases the MOT complexity. Therefore, we propose an object constraint learning to update these models adaptively. Our main idea is to update these models when the discriminability of those models is insufficient to differentiate tracked objects. To achieve this, we define an object constraint of the model discriminability and update the object models when inconsistency with the constraint occurs. In return, we can reduce the online learning complexity as well. We apply our global appearance and motion models, and the object constraint learning for the confidence-based data association [9]. We achieve the better performance than our baseline MOT method. We described our overall framework in Figure 1.

For a fair comparison, we evaluate our method on the public available MOT benchmark dataset, and provide extensive ablation studies and comparison results over state-the-of-the-art MOT methods. The experimental results prove the effectiveness of our methods.

To sum up, the main contribution of this paper for effective MOT can be summarized as follows:

- Proposition of the global appearance model for MOT using contrastive learning among tracked objects;
- Proposition of the global relation motion model for MOT using adversarial learning with object self motions and relative motions;
- Proposition of the object constraint learning to reduce the online learning computational complexity during the model update.

The rest of this paper is organized as follows: Section 2 discusses the related works with our proposed method. Section 3 describes our online multi-object tracking method with confidence-based object association and affinity models. Our global appearance model is proposed in Section 4. Section 5 presents our global relation motion model. Our object constraint learning for effective multi-object tracking is shown in Section 6. We provide our experimental results in Section 7. Finally, Section 8 concludes the paper.

## 2. Related Works

In this section, we discuss previous researches which are related to multi-object tracking. Firstly, online multi-object tracking (MOT) methods are introduced in Section 2.1. Then, we review the global appearance and motion model in Section 2.2 and Section 2.3, respectively. They are closely related to our proposed multi-object tracking method.

### 2.1. Online Multi-Object Tracking

Under tracking-by-detection paradigm, online tracking methods perform tracking with detections up to the current frame [9,10,11,12,13,14,15]. Therefore, online methods can be applied for real-time systems (e.g., ADAS, autonomous driving). However, association failures by occlusions occur more easily compared to batch methods [2,6,28,29]. Therefore, many methods focus on improving the data associations by learning affinity models [30]. To this end, appearance, motion, and shape models are exploited as object affinity models [4,9,23,31,32]. Depending on sharing an affinity model or not, we can categorize multi-object tracking into object-specific methods and global object methods. We provide the details of each method in Section 2.1.1 and Section 2.1.2, respectively.

#### 2.1.1. Multi-Object Tracking with Object-Specific Models

Object-specific models are usually generated by learning each object affinity model and use it for affinity evaluation or association between tracks or detections. Ref. [32] proposes an object-specific appearance learning based on appearance discriminability measures and a partial least square (PLS)-based subspace learning. In particular, the appearance and motion models are considered as core affinity models when distinguishing objects.

In addition, several multi-object tracking methods [14,15,18] exploit single object tracking (SOT) to learn object-specific features or models. Ref. [15] applies SOT with the attention mechanism. Ref. [13] learns short-term and long-term cues for addressing ID switches with a SOT and re-identification sub networks. Ref. [11] uses three independent CNN models for capturing appearance changes of objects more accurately. Ref. [19] utilizes two ResNet-50 networks to extract appearance and motion features. However, learning each object model is rather inefficient because computational complexity is proportional to the number of objects in the sequence. Ref. [33] presents an online multi-object tracking method with target-specific metric learning and motion dynamics estimation for the association.

#### 2.1.2. Multi-Object Tracking with Global Models

One of key ideas to enhance the tracking efficiency is to exploit global object models. The global models discriminate appearance and motions of tracked objects [4,9,22,24,25,32,34]. AP_RCNN [35] exploits features from a CNN-based detector as an global appearance model. Famnet [24] introduces an affinity sub-network to fuse discriminative higher-order appearance and motion information for the affinity evaluation. In detail, a feature sub-network is exploited for extracting features for target objects in an image frame, and an affinity sub-network estimates the higher-order affinity. They present the multi-dimensional assignment sub-network to find the global optimal assignments. Ref. [25] exploits a compressed deep CNN feature-based correlation filter tracker to exploit geometric and semantic information for the data association. They use ConvNet-based correlation filter (CCF) to make the detector generate more accurate bounding boxes.

These global object models alleviate the computational burden of learning object models during tracking. However, developing efficient multi-object models is still challenging due to the trade-off between tracking accuracy and speed.

To achieve effective object tracking, we also aim at learning global appearance and motion models. By exploiting these global object models, we can extract high discriminability appearance features and long-term future motions independently. We also design the suitable affinity models based on our global appearance and the motion features to enhance the data association during multi-object tracking. In addition, our object constraint learning reduces the online learning complexity of the global models efficiently because the models are updated according to their discriminability.

### 2.2. Global Appearance Model Learning

As discussed in Section 2.1.2, the global appearance model shows more effectiveness compared to the object specific model. However, this global model shows the lower discriminability power than the object specific model. Therefore, improving the model discriminability is a key for accurate association. To this end, a CNN-based feature [35,36,37,38,39] is often exploited as an appearance model due to its rich representation. Refs. [9,39] further enhance a CNN feature by using Siamese networks [40]. Ref. [36] uses a modified triplet loss of Siamese networks for extracting more robust features. MOTS R-CNN [37] develops cosine-margin-contrastive and cosine-margin-triplet losses to improve the appearance feature discriminability power. GTREID [38] proposes a graph neural-network-based multi-object tracking framework. They exploit a class-based triplet loss in order to extract the robust appearance features.

Some contrasting learning methods [41,42,43] are proposed to enhance self-supervised learning performance with the contrastive learning [42] aims to tackle background the bias problem in contrastive learning. Ref. [43] introduces a self-supervised objective trained with contrastive learning in order to disentangle object attributes from unlabeled videos. Ref. [41] proposes contrastive learning method between the global image and the local patch. They aim to learn consistent representation to enhance object-detection performance. The difference between them and ours is we learn the appearance feature which can identify the object, and utilize it for multi-object tracking directly. However, they only aim to consistent representation to object detection performance.

In this work, we learn the global appearance model via the contrastive learning with triplet loss. We use a lightweight ConvNet to extract high-level semantic features of tracked objects efficiently. For association, we extract appearance features of tracked objects from our global appearance model and compute the appearance affinity score by comparing those features. Furthermore, our object constraint learning encourages the reuse of the appearance model at most while the appearance model keeps its discriminability. As a result, we can improve the MOT accuracy and the tracking speed together.

### 2.3. Motion Model Learning

Since the appearance model can be contaminated by appearance changes or occlusions, learning motions of tracked objects is also important when predicting their motions or evaluating motion affinities. However, it is still challenging to learn the object motion accurately because of the abrupt camera motion changes or frequent occlusions by other objects. To address this, there are many studies to learn multi-object motions.

The Kalman filter [44] is mostly adopted as an object motion model. It predicts the current object state based on the previous states. Because of its high efficiency and flexibility, many multi-object tracking methods still exploit it to learn object motions [45,46]. Optical flow is also widely used for multi-object tracking as an object trajectory prediction method [47]. Among several traditional optical flow algorithms, Lukas–Kanade algorithm [48] is frequently used for the motion prediction and object tracking. However, these traditional methods are prone to under-perform under large motion videos. To achieve robust accurate optical flow results, several works introduce deep neural networks. FlowNet [49,50] predict dense optical flows of given images by using an encoder–decoder architecture network and a correlation layer. PWC-Net [51] combines traditional optical flow methods, such as image pyramid, warping, and cost volume with an end-to-end trainable deep neural networks. RAFT [52] uses multi-scale 4D correlation volumes and a recurrent unit to estimate optical flows in the video.

Furthermore, trajectory estimation methods [53,54,55,56,57,58] are suggested. They estimate the future trajectories by considering the relations between each objects. The predicted trajectories are useful for improving crowded scene tracking. Compare to the Kalman filter, the additional benefit is that learning object motions every frame is not required.

In studies of [55,56], they use a LSTM model to predict the motion trajectory with the relation between each pedestrian. Social-STGCNN [57] predicts the future trajectories by building a spatial-temporal graph using observed trajectories. Social-NCE [58] adopts contrastive loss in order to encourage keeping the positive event information from the negative information.

In our work, we adopt the trajectory estimation method [56] as our the global relation motion model in our MOT framework. To this end, we train this model on multi-object tracking datasets [59] rather than training it on trajectory estimation datasets [54,60]. We use this as our global motion model. Since it is difficult to capture local motion details of each object using the global motion model, we use an additional self-motion model to resolve this.

## 3. Online Multi-Object Tracking

In this section, we discuss our online multi-object tracking method. As mentioned in Section 1, we choose the confidence-based object association algorithm [9] as our baseline due to following reasons:

(1) Recent multi-object tracking methods tend to improve the accuracy by applying well-designed detection methods. For example, Refs. [2,28,61] exploit the Faster R-CNN head [62] which is one of popular detection methods. By using the detection method, they refine public detections by discarding false detections or correcting misaligned detections before feeding it to multi-object tracking network. Moreover, Ref. [63] attaches an appearance embedding feature head into a detector [64] in order to identify the tracked object, as well as more accurate object localizations compared to original public detections. They can improve the MOT accuracy but the overall tracking speed degrades in return because of the computational cost for detection. On the other hand, the confidence-based object association algorithm exploits public detections without any manipulation and additional inputs by detection heads. To improve the accuracy, this method aims to enhance the association quality which is key for robust multi-object tracking regardless of the quality of object location by detection methods.
(2) The confidence-based object association algorithm is one of representative multi-object tracking methods which improves the tracking accuracy by applying adaptive association methods (i.e., local association and global association) according to confidences of tracked objects. However, their affinity models used for the association are somewhat outdated. Therefore, in this work, we present more powerful affinity global appearance (in Section 4) and motion models (in Section 5), and the constraint learning method (in Section 6) to update affinity models effectively. As a result, we can improve both tracking accuracy and speed considerably.

### 3.1. Confidence-Based Object Association

In this section, we introduce the confidence-based multi-object tracking [9] as our baseline data association method. Before discussion, we denote detections from an object detector at frame *t* as $z_{t}=\left[z_{x},z_{y},z_{w},z_{h}\right]$, where $z_{x},z_{y},z_{w},$ and $z_{h}$ are a *x* and *y* position, width and height of a box, respectively. We define a set of detection as $Z_{t}$ at a frame *t*. We denote $O^{i}$ as a tracklet, and it can be associated with $z_{t}$ at each frame. Therefore, a tracklet $O^{i}=\left\{z_{k}^{i}|1<t_{s}^{i}<k<t_{e}^{i}\leq t\right\}$ is a short trajectory between $t_{s}^{i}$ and $t_{e}^{i}$ which indicates start and end time stamps. Therefore, the main problem of the frame-by-frame online association is how to associate a $O^{i}$ with a $z_{t}^{i}$ originated from this tracklet.

For the confidence association, we can evaluate the confidence of each $O^{i}$ with its length and continuity, and the affinity with an associated detection as follows:

(1) $$\begin{array}{cc} C(O^{i}) & ⊧\left(\frac{1}{L}\sum_{k\in \left[t_{s}^{i},t_{e}^{i}\right],v^{i}\left(k\right)=1}A\left(O^{i},z_{k}^{i}\right)\right)\\ \; & \times \left(1-\exp\left(-\beta \cdot \sqrt{\left(L-\lambda \right)}\right)\right), \end{array}$$

where C is a confidence function for a tracklet $O^{i}$ and $v^{i}\left(t\right)$ is a binary function to represent an association event between $O^{i}$ and $z_{k}^{i}$. Thus, $v^{i}\left(t\right)=1$ means that an associated detection $z_{k}^{i}$ for object *i* exists at frame *t*, otherwise $v^{i}\left(t\right)=0$. $A(O^{i},z_{k}^{i})$ is the total affinity score computed by Equation (4). *L* is the length of the tracklet as $L=\left|O^{i}\right|$, and β is a control parameter relying on the accuracy of detector. $\lambda =t_{e}^{i}-t_{s}^{i}+1-L$ is the number of skipped frames in which the object *i* is missing due to occlusion by other objects or unreliable detection.

When the confidence scores of a tracklet is calculated by Equation (1), we perform local and global association adaptively according to its confidence. A tracklet $O^{i(high)}$ with high confidence can be regarded as a reliable tracklet. We determine the high confidence and reliable tracklet as follows: (1) a longer tracklet can be considerable a reliable tracklet rather than a shorter tracklet; (2) a tracklet less occluded can be more reliable due to lower track fragment; (3) if a tracklet has high affinity score with an associated detection, we consider it as a reliable tracklet. Otherwise, a tracklet with a low confidence $O^{i(low)}$ is regarded as an unreliable tracklet.

We then categorize all tracklets into high- and low-confidence tracklets, and we apply different association methods in each group. In the local association, we associate $O^{i(high)}$ with $z_{t}$ since $O^{i(high)}$ has a higher possibility to be associated with $z_{t}$. On the other hand, in global association $O^{i(low)}$ is associated with other tracklets or remained detections after the local association. The reason is that these have a lower possibility of being associated with detections due to the track occlusion. In addition, the global association between tracklets can link fragmented tracklets.

$O^{i(high)}$ is locally associated with a detection. When *h* tracklets with high confidence and *n* detections $Z_{t}={\left\{z_{t}^{j}\right\}}_{j=1}^{n}$ are given at frame *t*, we compute a local association matrix $S_{local}$ as follows:

(2) $$\begin{array}{c} S_{h\times n}^{local}=\left[s_{ij}\right],s_{ij}=-A\left(O^{i(high)},z_{t}^{j}\right),z_{t}^{j}\in Z_{t} \end{array}$$

As discussed, a tracklet with low confidence $O^{i(low)}$ is considered an unreliable or fragmented tracklet. Therefore, we link this fragmented tracklet with other $O^{i(high)}$ or a detection $z_{i}^{j}$. Here, $z_{i}^{j}$ should not be associated with any $O^{i(high)}$ in the local association. We assume that η non-associated detections (η≤n), and *h* and *l* tracklets with high and low confidence, respectively. To link $O^{i(low)}$, we conduct global association in consideration of the following three possible events. Firstly, when $O^{i(low)}$ is associated with $O^{i(high)}$, we denote an event A. If $O^{i(low)}$ is terminated, we denote an event B. Lastly, we denote an event C when $O^{i(low)}$ is associated with $z_{t}^{j}$.

Then, we define a global association matrix based on these association events as follows:

(3) $$\begin{array}{c} S_{\left(l+\eta )\times (h+l\right)}^{global}=\left(\begin{array}{cc} A_{l\times h} & B_{l\times l}\\ -\theta_{\eta \times h} & C_{\eta \times l} \end{array}\right), \end{array}$$

where $A=[a_{ij}]$ corresponds to the event A and $a_{ij}=-A\left(O^{i(low)},O^{j(high)}\right)$ is an association score evaluated with the affinity model Equation (4). The event B is modeled as $B=\mathrm{diag}[b_{1},\ldots ,b_{l}]$, where $b_{i}=1-C\left(O^{i(low)}\right)$ is the score to terminate $O^{i(low)}$. The event C is determined by $c_{ij}=-A\left(O^{i(low)},z_{t}^{j}\right)$.

By exploiting $S^{local}$ or $S^{global}$, we can determine optimal matching pairs in each matrix by using the Hungarian algorithm [65].

### 3.2. Affinity Model

For the evaluation of a track confidence Equation (1) and the confidence-based association Equations (2) and (3), evaluating affinities between objects is important. To compute affinities accurately, we exploit several object models. We define object models of a tracklet $O^{i}$ as $\left\{A,S,M\right\}$, where A,S, and *M* are appearance, shape and motion models, respectively. Using these models, we define a total affinity model as

(4) $$\begin{array}{c} A\left(u,v\right)=A^{A}\left(u,v\right)\cdot A^{S}\left(u,v\right)\cdot A^{M}\left(u,v\right), \end{array}$$

where u and v are a tracklet or a detection. The appearance affinity is defined as follows:   

(5) $$\begin{array}{cc} \; & A^{A}\left(u,v\right)=\exp\left(-\frac{1}{c_{A}}\cdot d\left(f^{u},f^{v}\right)\right), \end{array}$$

where $c_{A}$ (=47.5 in our experiment) is a hyper-parameter for tuning this affinity, and d(·) is a L2 norm to evaluate a feature distance. $f^{u}$ and $f^{v}$ are the appearance features of u and v, respectively. They can be extracted from a global appearance model as discussed in Section 4.

To compute an affinity score between objects with their motions, we propose a novel motion affinity $A^{M}\left(u,v\right)$ based on their self and relation motions as follows:   

(6) $$\begin{array}{cc} \; & A^{M}\left(u,v\right)=c_{M}A_{relation}^{M}\left(u,v\right)+\left(1-c_{M}\right)A_{self}^{M}\left(u,v\right), \end{array}$$

where $A_{self}^{M}\left(u,v\right)$ is an motion affinity with self motions of u and v. We first predict each object self-motion using a Kalman filter [44], and evaluate the motion difference as follows:

(7) $$\begin{array}{cc} A_{self}^{M}\left(u,v\right)= & N\left({\hat{b}}_{u}^{tail}+v_{u}^{F}\Theta ;{\hat{b}}_{v}^{head}\Sigma_{self}^{F}\right)\\ \; & \times N\left({\hat{b}}_{v}^{tail}+v_{u}^{B}\Theta ;{\hat{b}}_{v}^{tail}\Sigma_{self}^{B}\right), \end{array}$$

where $\hat{b}$ is an updated position by Kalman filter. To compute this, we use the spatial difference between the head (i.e., the first updated position) to tail (i.e., the last updated position) of u and v head with time gap Θ. $v_{u}^{F}$ is the forward velocity, which is calculated from the head to tail of u. Otherwise, the backward velocity $v_{v}^{B}$ is computed from the tail to the head of v. We use the Gaussian distribution function for evaluating the spatial distance affinity between the predicted positions with the velocity and updated positions.

In addition, we use the $A_{relation}^{M}\left(u,v\right)$ as an another motion affinity model based on our global relation motion model. In some object tracking (e.g., pedestrian, car, etc.), it is useful to exploit the relation motions caused by their interactions and group behavior. Since these relation motions between objects are not captured by the self-motion model but these are crucial for MOT, we exploit the relation motion for affinity evaluation, and learn a global relation motion model using generative adversarial networks (GAN) [56]. We provide the details of predicting each object motion by using this relation model in Section 5.

The main benefit of our global relation model is that we can predict motions of all the tracked objects by considering other object motions from a certain frame to the next $\Delta_{est}$ frames. Therefore, it is not necessary to learn this model per frame, and it reduces the motion inference complexity. To combine both self and relation motion models effectively, we introduce a weight parameter $c_{M}$. We calculate $c_{M}$ by considering the range of motion prediction $1\leq \Delta <\Delta_{est}$ of the relation model. When our global relation motion model estimates the future motion, we set Δ to 1. Δ increases until our global relation model predicts the new future motion again. We calculate $c_{M}$ as follows:

(8) $$\begin{array}{c} c_{M}=\frac{\left(\Delta_{est}-\Delta \right)}{\Delta_{est}}, \end{array}$$

when Δ is a lower, we assign a higher weight to the relation model than the self-motion model. The reason is that the accuracy of the estimated motions tends to be reduced as Δ is increased (We verify this from an experiment in Section 7.6).

This affinity model $A_{relation}^{M}\left(u,v\right)$ can be defined as follows:

(9) $$\begin{array}{cc} \; & A_{relation}^{M}\left(u,v\right)=N\left({\hat{Y}}_{u}^{\Theta };{\hat{b}}_{v}^{head},\Sigma_{relation}^{F}\right), \end{array}$$

where N is a Gaussian distribution, ${\hat{Y}}_{u}^{\Theta }$ is the updated position of *u* from a global relation motion model with consideration of motion relation. ${\hat{b}}_{v}^{head}$ is the first refined position of *v*, and  $\Sigma_{relation}^{F}$ is a connivance matrix. Here, we exploit the forward motion only to increase tracking speed.

In order to consider the discrepancy of object sizes, we use a shape model for affinity evaluation. The shape affinity is defined as follows:

(10) $$\begin{array}{cc} \; & A^{S}\left(u,v\right)=\exp\left(-c_{S}\cdot \left\{\frac{h_{u}-h_{v}}{h_{u}+h_{v}}+\frac{w_{u}-w_{v}}{w_{u}+w_{v}}\right\}\right), \end{array}$$

where *h* and *w* are height and width of u and v, and $c_{S}$ (=1.5 in our experiment) is a tuning parameter for shape affinity.

## 4. Global Appearance Model

To achieve a robust association, object appearance is an important cue. Especially, exploiting effective appearance models reduces association failures under occlusions and appearance changes. In this section, we discuss our global appearance model. As we mentioned in Section 1, we use a light ConvNet to extract high-level semantic object features for tracked objects. With identifications of tracked objects, we define positive and negative object feature pairs. Additionally, we exploit the triplet loss for minimizing the feature distance between positive pairs but maximizing that between negative pairs.

### 4.1. Deep Feature Extractor

**Figure 2:**
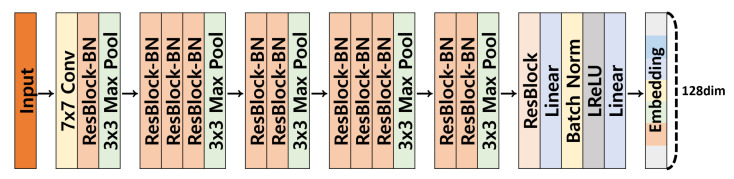
The architecture of our feature extractor. The ResBlock-BN means a resnet block bottleneck.

To extract the appearance features of objects, we use a modified ResNet-v2 network called LuNet [66]. The input of LuNet is an 128×64 image patch. This network uses LeakyReLU [67] as an activation function for a robust optimization as shown in [68], multiple 3×3 max-poolings with stride 2 instead of strided convolutions, and eliminates the final average pooling layer of feature-maps in the last res-block as depicted in Figure 2. From the multi-layer perceptron (MLP) layer of the last layer, we extract a 128-dimensional embedding feature of an object. This network is lightweight (5 M parameters) compared to other feature extraction networks (e.g., ResNet-50). We train this network with triplet loss [26] for learning discriminable features. We provide the details of our training method in the next section.

### 4.2. Triplet Loss

**Figure 3:**
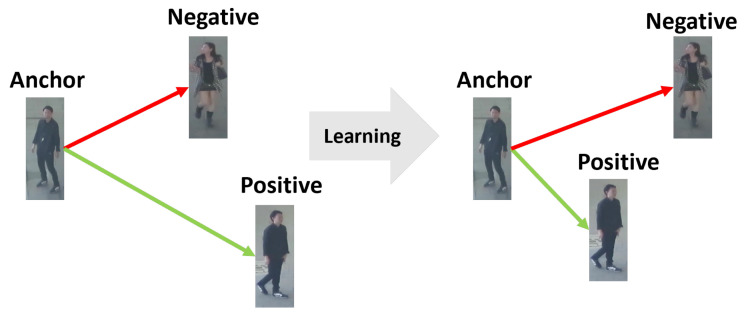
The change of object feature distances by using the triplet loss. The positive and the negative samples are connected with an anchor sample and indicated by green and red arrows, respectively.

In order to discriminate tracked objects in consecutive frames, it is important to compute the distance between object appearance features accurately. To this end, we train our global appearance model based on the triplet loss [26]. We define an anchor $x_{a}^{i}$, positive $x_{p}^{i}$, and negative $x_{n}^{i}$ objects with their IDs. The anchor means a targeted object. The positive object has the same ID with the anchor object, but the negative object has a different one. We can extract 128-dimensional embedding features by using the feature extractor f(·), and denote $f(x_{a}^{i})$, $f(x_{p}^{i})$, and $f(x_{n}^{i})$ as anchor, positive, and negative features. For increasing the discriminability between objects, we should minimize a distance between $x_{a}^{i}$ and $x_{p}^{i}$, whereas maximize that between $x_{a}^{i}$ and $x_{n}^{i}$, as shown in Figure 3. For achieving this, we can define a triplet loss as:

(11) $$\begin{array}{c} L_{triplet}=\max\left(0,m+d\left(f\left(x_{a}^{i}\right),f\left(x_{p}^{i}\right)\right)-d\left(f\left(x_{a}^{i}\right),f\left(x_{n}^{i}\right)\right)\right), \end{array}$$

where d(·) is the distance function between two embedding vectors and *m* is a margin to force the distance between positive and negative samples.

### 4.3. Online Hard Triplet Mining and Loss

Basically, the triplet loss is evaluated with distances of anchor/positive and anchor/negative pairs. Therefore, the sample pair selection largely affects this metric learning. To determine the meaningful sample pairs during online tracking, we use online triplet mining. We select possible triplets in each batch. Once embedding feature vectors are extracted for tracked objects from our feature extractor, we construct sample triplets with their object IDs. Based on different sample combinations, all sample features can be utilized as anchor, positive, and negative ones. We can select training sample triplets randomly in a batch, and use those for the metric learning Equation (11). However, for more effective learning, we present the online hard triplet mining. The basic idea is to use the hardest positive and negative sample based on anchor. Here, the hard positive one has the lowest affinity Equation (4) among positive samples in the batch. One the one hand, the hard negative one has the highest affinity among negative ones. In the worse case, the affinity of the positive pair is likely to be lower than that of the negative one. As discussed in [66], exploiting these hard triplets is more effective than random training sample selection in reducing the convergence time and enhancing the model accuracy.

**Figure 4:**
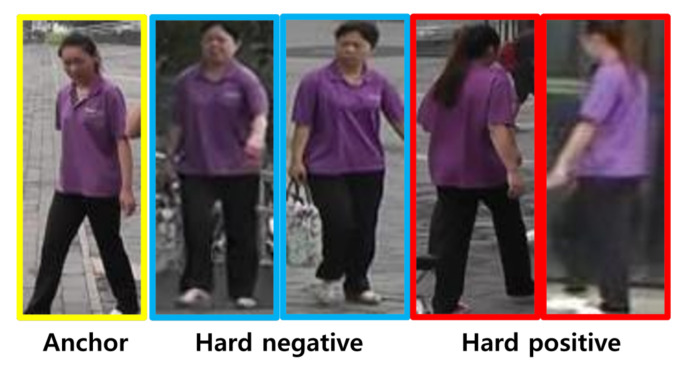
Examples of an anchor, hard negative, and hard positive samples.

Based on this idea, we can transform the conventional triplet loss Equation (11) into a hard triplet loss as:

(12) $$\begin{array}{cc} L_{hard}=\sum_{i=1}^{P}\sum_{a=1}^{K}[m & +\max_{p=1\cdots K}d\left(f\left(x_{a}^{i}\right),f\left(x_{p}^{i}\right)\right)\;\\ \; & -\min_{\begin{array}{c} j=1\ldots P\\ n=1\ldots K\\ j\neq i \end{array}}d\left(f\left(x_{a}^{i}\right),f\left(x_{n}^{j}\right)\right){]}_{+}, \end{array}$$

where ${\left[\cdot \right]}_{+}$ is a hinge loss, *P* is the number of distinguished object IDs, and *K* means the number of sample images per class. *i* and *j* are class indices (i≠j). The second and third terms represents positive and negative hardest sample selection, respectively. By using these difficult samples for training our model f(cot), we can improve the discriminability of our model further. We provide some instances for hard positive and negative samples in Figure 4. As shown in Figure 4, the hard negative one with different object IDs looks similar to the anchor because both persons wear the similar clothes. However, the hard positives for the same person seem to be different appearances due to the viewpoint changes.

## 5. Global Relation Motion Model

It is important to predict an object future trajectory accurately for achieving the high quality of MOT. As mentioned in Section 3.2, we use self and relative motion models for evaluating the motion affinity. In this section, we present our relative motion model and learning method in detail.

Considering the relative motion is one of the key ideas to estimate future trajectories more accurately because the motion of an object (e.g., pedestrians, obstacles, etc.) can be influenced by nearby objects and obstacles during tracking. Due to this reason, some recent studies estimate multi-object trajectories in consideration of the relative motion with non-linear model such as LSTM [55,56]. Therefore, we also use relative motions for estimating global motions for all tracked objects.

For estimating the future trajectory, our global relation motion model exploits consecutive $\delta_{obs}$ frames ($\delta_{obs}$ = 5 in our experiment). Therefore, an input of this model is a set of motion trajectories $X=\left\{X_{t-\delta_{obs}-1},\ldots ,X_{t}\right\}$ from previous $t-\delta_{obs}-1$ to current *t* frames, and the output is a set of estimated trajectories $\hat{Y}=\left\{{\hat{Y}}_{t+1},\ldots ,{\hat{Y}}_{t+\Delta_{est}}\right\}$ to the next $t+\Delta_{est}$ frames. $X_{t}$ and ${\hat{Y}}_{t}$ are tracked object trajectories consisting *x* and *y* coordinates for previous and future frames, respectively.

Our global relation motion model is trained based on the adversarial learning to understand distributions of various and relative motions. We explain the details of the adversarial learning in Section 5.1. Our model consists of two networks, the trajectory generator *G* and discriminator *D*. The generator *G* captures the distribution of the motion history and estimates future motions. The discriminator *D* computes the confidence score for ground truth trajectory Y or the predicted trajectory $\hat{Y}$. We provide the detailed structures of *G* and *D* in Section 5.2.

### 5.1. Generative Adversarial Networks

In this section, we first review the traditional generative adversarial networks [27] in brief before introducing our motion learning method. Basically, a generator and a discriminator are trained by exchanging the feedbacks of the other network. Therefore, a generator *G* tries to capture the sample distribution of a trained dataset and tries to generate more realistic samples for deceiving a discriminator *D*. Ideally, the distribution of generated samples is close to the distribution of real samples. A generator *G* takes a latent vector z as its input, and outputs the generated sample G(z). On the other hand, a discriminator takes the real sample from a trained set or the fake sample generated from a generator. The output of D(·) is the confidence score of a sample. Thus, the loss of this adversarial learning is usually represented based on the two-player min–max game as follows [27]:

(13) $$\begin{array}{cc} L_{adv}=\min_{G}\max_{D}\; & E_{x∼p_{data}\left(x\right)}\left[\log D(x\right)]+\\ \; & E_{z∼p_{z}\left(z\right)}\left[\log\left(1-D(G(z))\right)\right], \end{array}$$

where $p_{data}$ and $p_{z}$ are the distributions of the given dataset and the generated sample, respectively. We can extend this loss for the global motion learning. For trajectory generation, we define the following adversarial loss $L_{M}$

(14) $$\begin{array}{cc} L_{M}=\min_{G}\max_{D}\; & E_{Y∼p_{data}\left(Y\right)}\left[\log D(Y)\right]+\\ \; & E_{z∼p_{z}\left(z\right)}\left[\log\left(1-D\left(G\left(z,X^{*}\right)\right)\right)\right], \end{array}$$

where Y is the ground truth motion, and $X^{*}$ is the selected trajectory with the highest precision over Y).

One of main difficulties of training a global relation motion model is the unstable motion estimation due to the limited samples (i.e., trajectories). In fact, the object motions are too diverse, but the collected trajectories during tracking are very limited to handle all cases. To encourage the motion models to predict diverse motions, we randomly sample the latent vector z with N(0,1) for $N_{T}$ times ($N_{T}=20$ in our case). Therefore, we can generate $N_{T}$ trajectories per one sample in the generator. Even though we can use all the generated samples for training, we use Top-1 sampling for handling the sensitivity issue. We choose the closest one $X^{*}$ to a real trajectory, and the selected one for the adversarial learning Equation (14). By using the Top-1 sampling, we can increase the diversity of predicted motions rather than using all the generated samples.

### 5.2. Generator and Discriminator

**Figure 5:**
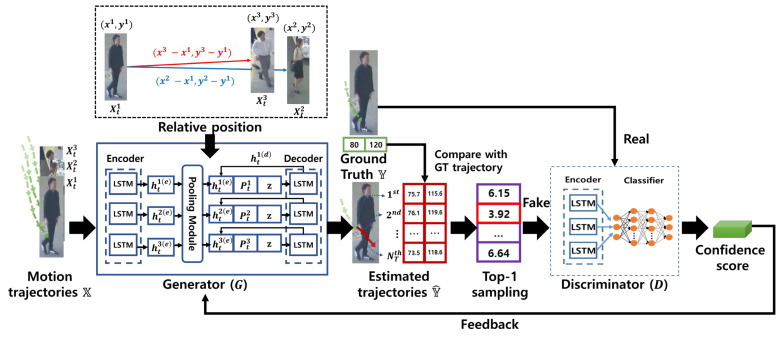
The architecture of our global relation motion model.

In this section, we discuss the structures of the generator *G* and the discriminator *D*. We describe the overall architecture of our global relation motion model with a generator *G* and a discriminator *D* in Figure 5. The details of each network are explained as below.

#### 5.2.1. Generator

In order to estimate motions of each object during online tracking, the generator *G* is exploited. We construct X with *x* and *y* coordinates of all tracked objects during multi-object tracking. The generator *G* consists of three networks: an encoder, a pooling module, and a decoder. The details of each network are explained as below.

**Encoder:** From the encoder, we can learn hidden states $h_{t}^{i(e)}$ of each object *i* at current frame *t* with its previous motions and hidden states. An encoder extracts a motion embedding vector with a fully-connected layer with LeakyReLU, and outputs hidden states $h_{t}^{i(e)}$ of an object *i* with a LSTM [69]. Then, we feed the learned hidden state $h_{t}^{i(e)}$ to the pooling network.

**Pooling module:** To consider relative motions between objects, we use a pooling module. The pooling module first calculates relative positions between an object and other different objects. These relative positions are calculated by subtracting *x* and *y* coordinates of targeted object and other objects. They are concatenated with each object’s hidden states. Then, they are embedded by a MLP with LeakyReLU independently. Lastly, embedded vectors are pooled (we use max-pooling in our research) to calculate a pooling vector $P_{t}^{i}$ of each object *i*. By using a pooling vector $P_{t}^{i}$, we can summarize relative information, and use it to predict the future trajectory in a decoder.

**Decoder:** The decoder consists of two MLPs and a LSTM network. As an input of this, we use the outputs of the encoder and pooling module. We concatenate the hidden state $h_{t}^{i(e)}$, the pooling vector $P_{t}^{i}$, and a latent vector z. This feature represents the object motion $h_{t}^{i(e)}$ and the relation motion between the object and other objects $P_{t}^{i}$. Then, z is embedded additionally to estimate *i*-th object feature motions in consideration of the object’s direction and speed with learned motion distributions. The concatenated features are passed into the LSTM of the Decoder, and this network outputs a hidden state $h_{t}^{i(d)}$. The last MLP predicts future trajectories of each object using $h_{t}^{i(d)}$. Then, we concatenate $h_{t}^{i(d)}$, $P_{t}^{i}$, and z. Then, we feed the concatenated feature to the LSTM iteratively until the decoder predicts $\Delta_{est}$ trajectories.

#### 5.2.2. Discriminator

The discriminator *D* consists of an encoder and a MLP as a classifier. The input of *D* is a ground truth trajectory (i.e., real) and predicted trajectory (i.e., fake) by the decoder. Then, an encoder of *D* extracts hidden features for the input trajectory similar to the encoder of *G*. It then predicts hidden states recursively to refine them more. By feeding the last hidden state of the encoder into a fully-connected layer, we can predict the classification confidence whether the input trajectory is a real one or not. The confidence of *D* is used for computing the $L_{M}$ Equation (14).

## 6. Object Constraint Learning

In this section, we introduce our object constraint learning method for controlling the update schedule of our global appearance model. As we discussed in Section 1, online learning of our global models at every frames increases the MOT complexity. We assume that the appearance features have enough discriminability to evaluate the affinity between objects at future frames. Based on our hypothesis, we can enhance the tracking speed and accuracy simultaneously by updating models adaptively according to discriminability of object models. To this end, we propose an object constraint learning in this section. Our idea is simple and easy to implement. We only update our model when inconsistency with the constraint occurs.

**Figure 6:**
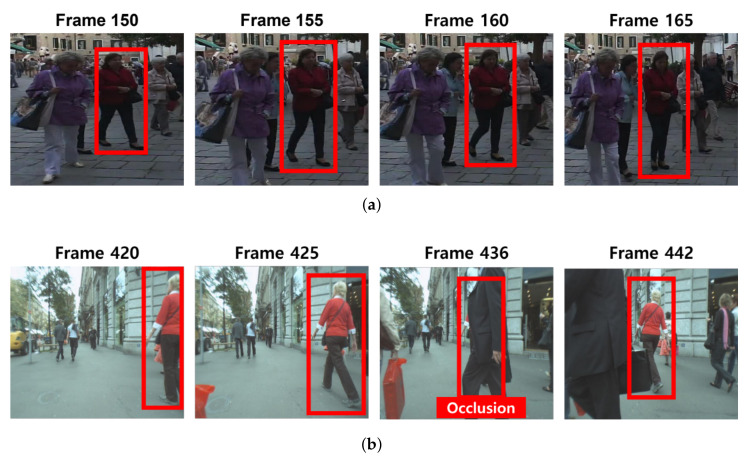
Examples of appearance changes in two different sequences. (**a**) Low appearance variation of a tracked object during tracking. (**b**) Abrupt appearance variation of an occluded object at frame 436.

To reduce the number of updates for our global appearance model, we consider the discrimiability of appearance features. We use the correlation of the object appearance between consecutive frames for calculating discriminability of the appearance feature. If objects has no abrupt appearance change during several frames, the appearance features extracted at these frames are similar. Therefore, we can utilize the appearance feature of the past frame for tracking at the current frame. However, when the features have a low similarity because of appearance change or occlusion, we should update features in the current frame. We describe these situations in Figure 6. As shown, the highlighted pedestrian within the red box in Figure 6a shows low appearance variation at frames. In this case, it is not necessary to update the object appearance model because the appearance feature extracted at 150 frame has enough discriminability to distinguish the same object at 165 frame. On the other hand, the highlighted pedestrian within the red box in Figure 6b shows the drastic appearance change at 436 frame because of the occlusion by other pedestrian. In this case, we need to update our appearance model at 442 frame to distinguish an occluded object.

To measure discriminability of an appearance model at frame *t*, we calculate $\rho_{t}$ with the appearance affinity $A^{A}\left(O^{i},O^{j}\right)$ between two different tracklets $O^{i}$ and $O^{j}$. We define $\rho_{t}$ as follows:

(15) $$\begin{array}{c} \rho_{t}=1-\frac{1}{N_{t}^{2}-N_{t}}\left(\sum_{i=1}^{N_{t}}\sum_{j\neq i}^{N_{t}}A^{A}\left(O^{i},O^{j}\right)\right), \end{array}$$

where $N_{t}$ is the number of tracklets at frame *t*. Using Equation (15), we compute the average appearance affinity score. To set $\rho_{t}$ in [0,1], we conduct min–max normalization for each $A^{A}\left(O^{i},O^{j}\right)$.

If $\rho_{t}$ is close to 1, we consider our global appearance model has the sufficient discriminability to distinguish objects. We set a threshold μ to 0.6 for our experiment. μ is tuned manually for high-quality multi-object tracking by considering tracking accuracy and tracking speed together. When $\rho_{t}>\mu$, we do not update the appearance model, because our model still maintains high discriminability. Otherwise, we update the model at frame *t*. In addition, we update object appearances when a new object appears or an existing track is terminated. To sum up, we update our global appearance model based on the following two constraints:

- Constraint 1: $\rho_{t}$ becomes lower than μ;
- Constraint 2: $N_{t}\neq N_{t-1}$.

For ease of implementation, we present Algorithm 1 for our object constraint learning with the global appearance model. In addition, we provide the overall multi-object tracking algorithm based on our proposed global model and object constraint learning in Algorithm 2. 
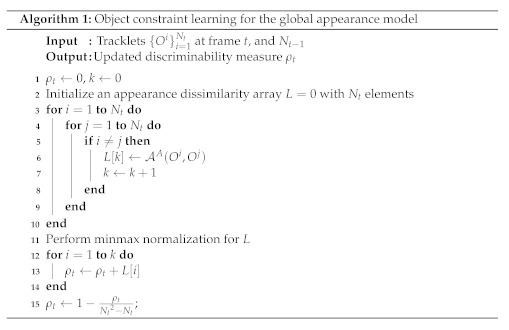
 
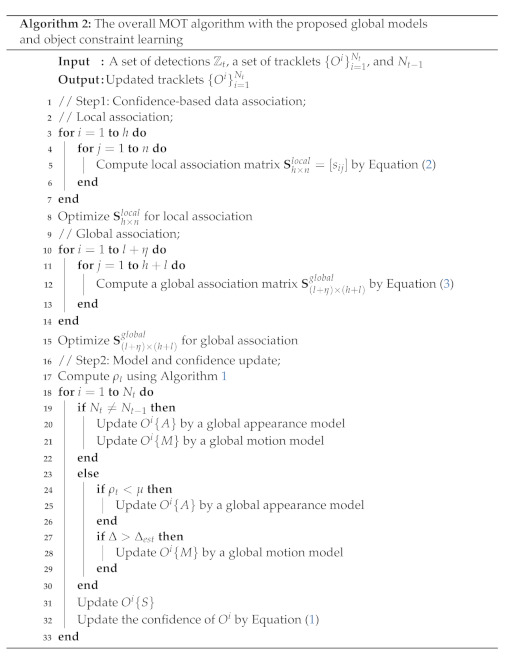


## 7. Experimental Results

In this section, we verify the effectiveness and benefits of our proposed method. We compare our method with other multi-object tracking methods and make ablation studies on MOT challenge datasets.

### 7.1. Datasets

To prove the effectiveness of our method, we exploit the 2016 multi-object tracking challenge benchmark dataset (MOT16) [70] for pedestrian tracking. This dataset consists of 7 training and 7 test sequences with different video frame rates captured by static or dynamic cameras. Additionally, they are captured from various locations (e.g., a large square, day/night street scene, and busy shopping mall and intersection) and viewpoints (e.g., elevated viewpoint, front viewpoint, and side viewpoint). Furthermore, the crowded density of objects is different from each other. To compare on the fair and same circumstance, we only exploit public detections and ground truth provided in the MOT16 challenges.

For training our global appearance model, we use the Market-1501 [71] dataset, which is constructed to handle a person re-identification problem. This dataset has person location information as bounding boxes from a person detector. This set contains 32,668 images of 1501 people. Then, this set is divided into training and test sets of 12,936 and 19,732 images for 750 and 751 persons, respectively. For training our global appearance model, we exploit the training set as done in [71].

**Figure 7:**
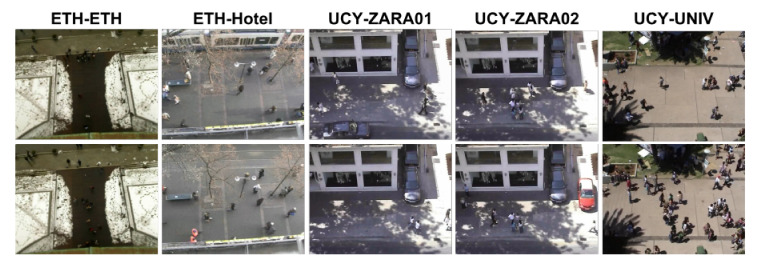
Examples of ETH and UCY datasets.

To learn our global relation motion model, we exploit ETH [54], UCY [60], and MOT15 datasets. They represent pedestrian trajectories in real world coordinates. Thus, this dataset provides the frame number, person ID, and x,y, and *z* positions per image. As shown in Figure 7, this dataset contains videos captured only from top-view and statics cameras. For improving the robustness of our global relation motion model over geometric motion variations, we use the MOT15 dataset which contains sequences from various and dynamic viewpoints. The MOT15 dataset consists of 11 training and 11 test sets. For training, we use 7 training sets which are not overlapped with the MOT16 dataset.

### 7.2. Implementation Details

Basically, we have implemented our MOT system based on Algorithm 2. We have tuned all hyper-parameters for the confidence-based multi-object tracker from the empirical search. However, the determined parameters are fixed for all evaluations. We use C++14 and the Armadillo library [72].

The network of our global appearance model outputs 128-dimensional embedding vectors from the input object images. We tune the appearance embedding feature dimensionality to 128 by considering both MOT accuracy and speed as shown in Section 7.5.3. We set the mini-batch for training the appearance model by using the online triplet mining [26] as mentioned at Section 4.3. In our mini-batch, we use 4 images for 32 different persons. Thus, the mini-batch size is 128. We tuned the margin *m* to 0.6 in Equation (11). The Adam optimizer with $\beta_{2}=0.9$ is used. When training the global appearance model, we set the initial learning rate to $5\times 10^{-4}$.

We also train the discriminator *D* and generator *G* for our global relation motion model. The mini-batch for training this model includes GT object trajectories during $\delta_{obs}+\Delta_{est}$ frames. In this experiments, we tuned the observation range $\delta_{obs}$ and prediction range $\Delta_{est}$ to 5 and 8, respectively. We use the Adam optimizer with $\beta_{2}=0.999$ to train the discriminator *D* and the generator *G*. We set the initial learning rate of *D* and *G* to $10^{-4}$ and $10^{-3}$, respectively. We exploit the gradient clipping method for training *D* and *G* to avoid gradient exploding, and set threshold for gradient clipping to 0.2 in order to prevent the model divergence during training. We use PyTorch [73] for implementation of our global relation motion model.

All our experiments are conducted on a single NVIDIA TITAN Xp GPU and an Intel i7-8700K CPU.

### 7.3. Performance Evaluation Metrics

To measure the multi-object tracking performance, we use metrics used in the MOT benchmark challenge. The details of the metrics can be found in [74]. We use the following metrics: multi-object tracking accuracy (MOTA ↑), multiple object tracking precision (MOTP ↑), ID F1 Score (IDF1 ↑), the ratio of mostly tracked trajectories (MT ↑), the ratio of mostly lost trajectories (ML ↓), the number of false positive (FP ↓), the number of false negative (FN ↓), the number of identity switches (ID Sw. ↓), and multi-object tracking speed (HZ ↑). ↑ and ↓ represent higher and lower scores, respectively.

MOTA score is widely exploited to measure the accuracy of multi-object tracking methods. MOTA is calculated as follows [70]:

(16) $$\mathrm{MOTA}=1-\frac{\sum_{t}\left(FN_{t}+FP_{t}+IDSW_{t}\right)}{\sum_{t}GT_{t}},$$

where, *t* is the frame index. $GT_{t}$, $FN_{t}$, $FP_{t}$, and $IDSW_{t}$ mean that the number of ground truth, false negative, false positive, and ID switch at frame *t*, respectively. As shown in Equation (16), FP, FN, and ID Sw. are considered the important metrics to calculate tracking accuracy. Note that, ID Sw. occurs when tracked identity is different with its matched ground truth identity [75]. MOTP is a metric which indicates the average dissimilarity between every true positives and their corresponding ground truth [70]. IDF1 score indicates the ratio of correctly identified detections over the average of ground truth and computed detections. MT and ML are employed to measure the the tracking methods cover the ground truth trajectories by predicted track. If the predicted track covers at least 80% of ground truth, it is regarded as mostly tracked (MT). On the other hand, it is considered as mostly lost (ML) when it covers less then 20% of ground truth.

### 7.4. Comparison on the MOT Benchmark Challenge

To compare with other state-of-the-art MOT methods, we evaluate our MOT method on the MOT benchmark challenge website. In Table 1, we show the evaluation results of our and other MOT methods. For the fair evaluation, we only use the public detections provided by the 2016 multi-object tracking challenge. For reliability, we present the scores of MOT methods that have achievements opened in journals or conferences. We exploit our global models and shape model for calculating affinity scores, and object constraint learning algorithm for enhancing tracking speed. Additionally, we apply our object constraint algorithm for all sequences. Our proposed method shows better multi-object tracking accuracy and speed than [5,19,32,76]. Refs. [11,13,14,15,29] show higher MOTA scores, but much lower tracking speed than our proposed method. In addition, our proposed method shows a lower number of ID switches than [5,11,14,15,19,32,76,77,78,79,80]. t represents that our proposed global models can improve the data association quality.

Note that our proposed method has higher tracking speed than most multi-object tracking methods. As mentioned, our object constraint learning indeed contributes to reduces the number of model updates. Even though the speed of [77,81] is faster than ours, but our method is superior to them in terms of the accuracy. For the tracker-level comparison, we also refine the original public detection using the CenterNet and then feed them to our tracker, as done in other trackers [2,28,61,81,82] As shown in Table 1, our method shows 64.5% MOTA and 6.54 Hz tracking speed which are competitive scores compared to recent published tracking methods in the MOT16 benchmark. This result indicates that our tracker with the global affinity models and object constraint algorithm indeed achieves a high-quality MOT.

### 7.5. Ablation Studies

#### 7.5.1. Comparison with the Baseline MOT Method

We compare our proposed method with our baseline confidence-based MOT [9] to verify the effectiveness of our global models. For fair comparison, we only use the 2016 MOT challenge train dataset. Appearance and motion models of the baseline are a 144-dimension color histogram feature and Kalman filter, respectively. This appearance model extracts appearance features from image patches, and computes similarity distance using the Bhattacharyya distance. The motion model of the baseline only uses Equation (7) as mentioned in Section 3.2. The baseline MOT and our proposed methods use the same shape model. In this ablation study, we do not exploit our object constraint learning method to verify the effectiveness of our affinity model learning method.

The comparison result is shown in Table 2. Our proposed MOT method shows an improved tracking accuracy for the most metrics. We improve MOTA and MOTP scores by 3.21% and 0.79%, respectively. Our proposed method also suppresses FP, FN, and ID switch than baseline due to the higher appearance discrimiability than the color histogram based appearance model. The baseline shows the faster tracking speed than our proposed method. Because their models have lower computational costs than ours. However, when considering the trade-off between tracking accuracy and speed, our method is more competitive than the baseline. To sum up, adding our global models to the baseline increases the MOT accuracy significantly without drastic decrease in tracking speed.

#### 7.5.2. Global Object Model Comparison

To prove the effectiveness of our global object models in terms of MOT accuracy, we implement different versions of multi-object tracking methods with/without our global models. The description of the implemented methods with our global models are given as:

(M1) Baseline multi-object tracking method;
(M2) Combining global relation motion model with M1;
(M3) Combining global appearance and global relation motion models with M1.

For fair comparison, we only use the 2016 multi-object tracking benchmark challenge train set (MOT16 train set), and the same confidence-based data association method with same hyper parameters. Additionally, the object contraint learning is not exploited for this comparison. The comparison results are presented in Table 3. Color and deep represent the color histogram appearance model and our global appearance model, respectively. The motion models are divided as self and relation models as we described in Section 3.2.

When comparing (M1) with (M2)–(M3), the baseline reduces the the MOT accuracy. The most metric scores except for ML decrease. Note that, the comparison result between (M1) and (M2) shows that our global relation motion model contributes to enhancing MOT results. In particular, our proposed motion model reduces FP, FN, and ID switch successfully. This result shows that using self and relation motions is more effective for improving trajectory estimation than using the self motion only. (M3) shows the best MOT performance among these methods. Especially, ID switch of (M3) decreases considerably compared to (M2). It shows that our global appearance model handles appearance changes better which is often caused by occlusion. To sum up, this experiment proves that our proposed global models can improve the quality of MOT results.

#### 7.5.3. Appearance Model Comparison

Table 4 shows the comparison results of appearance models with different embedding feature dimensions. As shown, extracting a 64-dimensional embedding feature shows better accuracy compared to others. However, we need to consider both accuracy and speed in high-quality multi-object tracking. In order to find out the best sweet spot [32], we compare multiplied scores of MOTA and Hz. As a result, we find that extracting 128-dimensional embedding features show the better score than others. From this comparison, we set the dimension of the embedding vector to 128.

#### 7.5.4. Motion Model Comparison

We show the effectiveness of combining our relation motion and self motion models. The self motion model only exploits a Kalman filter [44] and Equation (7) for calculating the motion affinity. The relation motion model uses only our global relation motion model in Section 5 and Equation (9) for computing a motion affinity score. Lastly, the combined motion model utilizes both motion models and Equation (6) to calculate the motion affinity.

The result is shown in Table 5. Comparing with self motion model and relation motion model results, the self motion model shows slightly a better tracking accuracy. The one possible reason is the future trajectory estimation results have possibility to be discordant due to abrupt motion and relation changes. However, our global relation motion model has a distinct advantage that is not necessary to learn and update this model per frame as we mentioned in Section 3.2. Therefore, we can ensure the improvement of tracking speed. We prove the advantage of our global relation motion model in Table 6.

The combined motion model shows higher multi-object tracking accuracy for the most metrics. This result represents that using combined self and relation motions is effective for improving multi-object tracking. The ID Sw. number of the combined model is higher slightly than other motion models. However, we exploit the advantages of each self and relative motion models by combining them for the data association. In addition, our weight parameter $c_{M}$ controls the weights of self and relation motions appropriately when evaluating the motion affinity Equation (6). As a result, we show that our proposed combined motion models with the weight parameter improve multi-object tracking accuracy the most.

#### 7.5.5. Object Constraint Learning

To prove the effectiveness of our object constraint learning introduced in Section 6, we compare MOT methods with/without our object constraint algorithm. For fair comparison, we use MOT16 train set, and the same confidence-based data association method with same hyper parameters. We set μ to 0.6 as we mentioned in Section 6.

Table 6 shows the results. The MOTA scores are improved by 0.69% when not using the object constraint learning. Other metric scores, such as IDF1, MT, FP, FN, and ID switch, also increase. However, we can obtain an almost similar accuracy by using our constraint learning although not updating models at every frame.

For the tracking speed, our learning algorithm shows the obvious gain since the number of appearance model updates deceases prominently. As shown in the table, the update number decreases dramatically when applying our learning algorithm. In particular, MOT16-05 (837 → 334), MOT16-09 (525 → 193) and MOT16-11 (900 → 255) sequences show the results. Therefore, we confirm that our object constraint learning determines the timing for model update successfully based on model discriminability.

### 7.6. Qualitative Results

**Figure 8:**
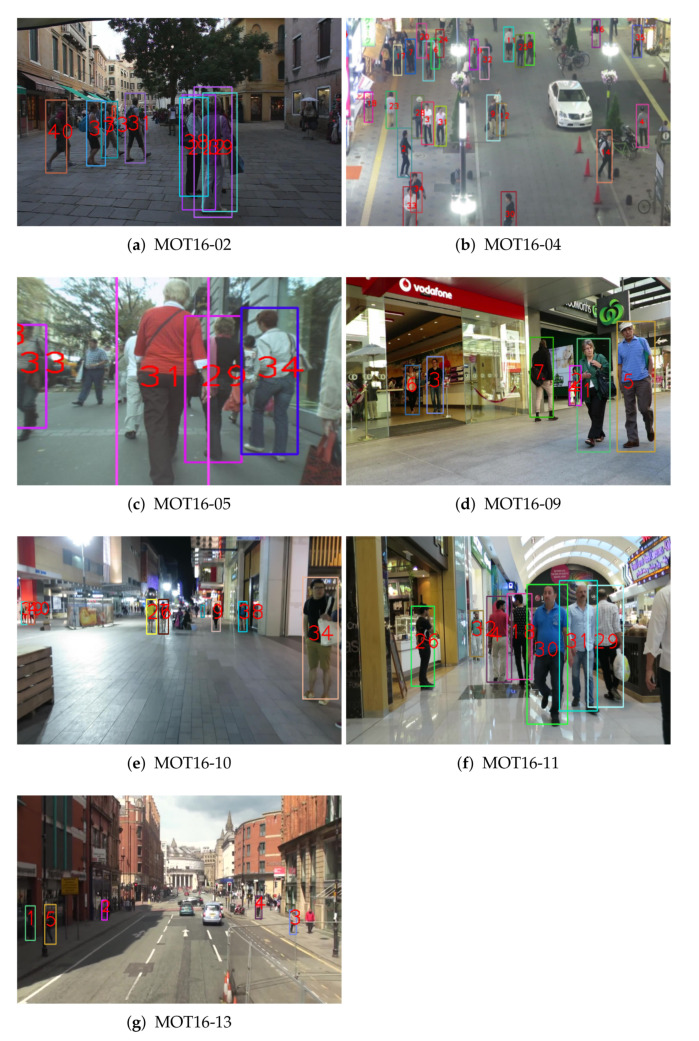
(**a**–**g**) Tracking results using the proposed MOT method with global appearance and motion models on the 2016 MOT benchmark train dataset.

**Figure 9:**
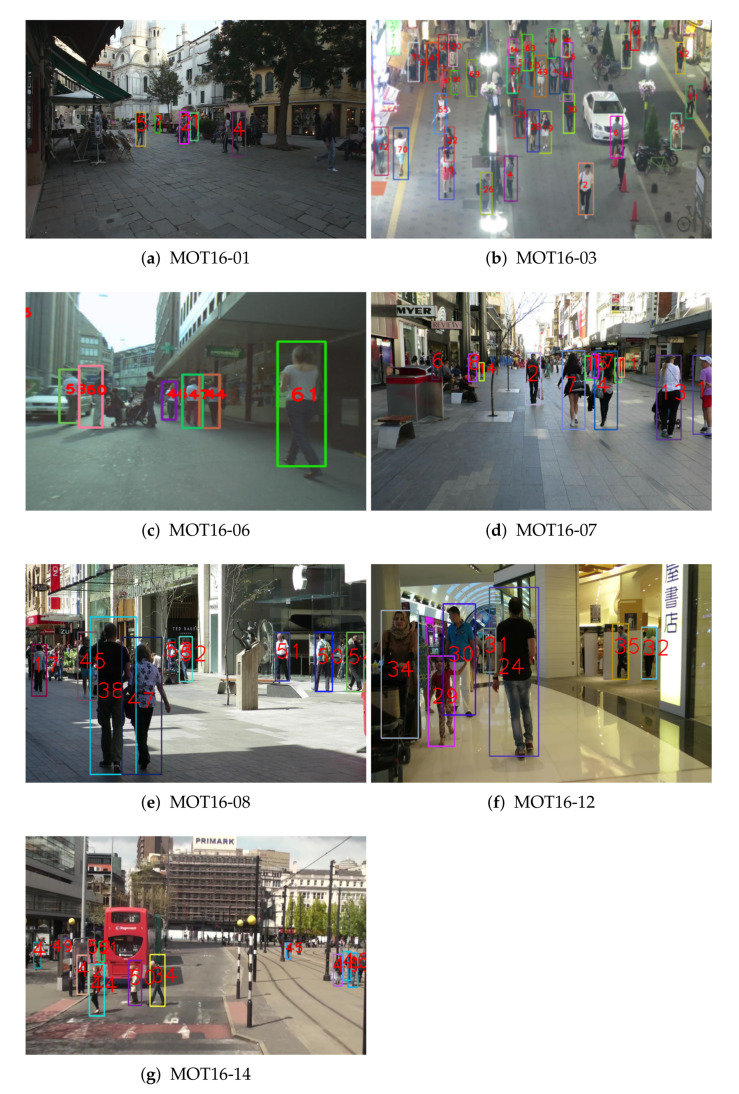
(**a**–**g**) Tracking results using the proposed MOT method with global appearance and motion models on the 2016 MOT benchmark test dataset.

Figure 8 and Figure 9 show the tracking results from our proposed global appearance and motion models on the 2016 MOT benchmark train and test dataset, respectively. Our proposed method successfully conducts multi-object tracking. Especially, our proposed method can track objects robustly in crowded and occluded sequences, such as Figure 8b and Figure 9b.

**Figure 10:**
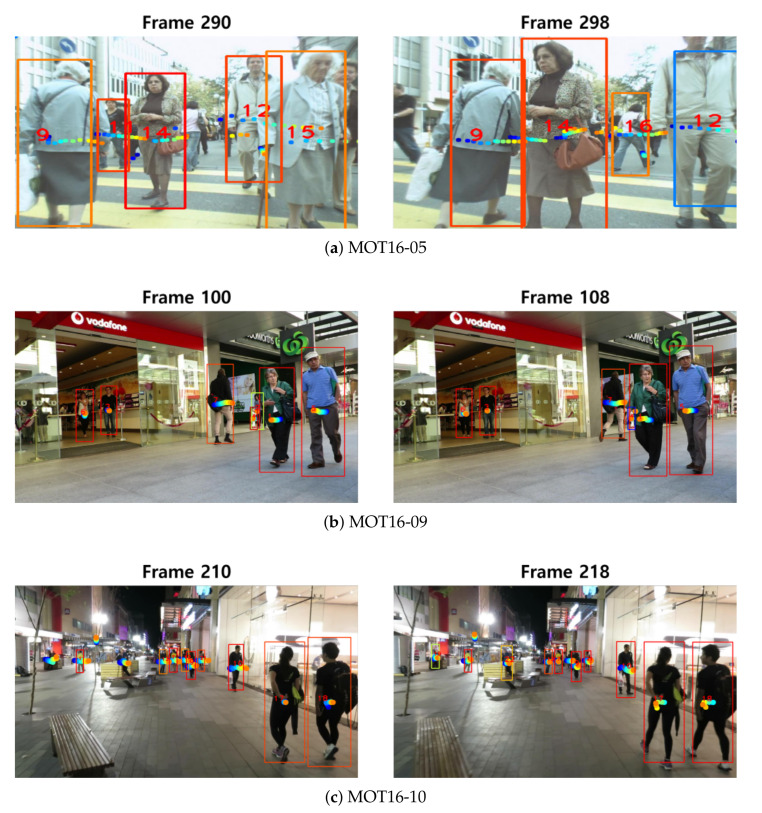
Predicted object motion trajectories on several sequences. At current frame *t*, the predicted motions from t+1 (blue) to $t+\Delta_{est}$ (orange) are depicted with different dot colors on each sequence.

Figure 10 describes the motion trajectory estimation results on the 2016 benchmark dataset. Even though several scenes are captured with low frame rates and with a moving camera (e.g., MOT16-05 and MOT16-10), our global relation motion model can estimate accurate object trajectories during multi-object tracking.

## 8. Conclusions

In this paper, we have proposed an effective multi-object tracking method by using the proposed global appearance and motion models based on our object constraint learning algorithm. As a result, our global object models successfully improve the tracking accuracy since they demonstrate the high appearance discriminability and accurate trajectory estimations. In addition, our object constraint learning algorithm alleviates the computational costs of learning object models in online. Based on the proposed methods, we can enhance tracking accuracy and speed together. Moreover, our global appearance and motion models can be compatible with other multi-object tracking methods because they do not rely on system architecture. The object constraint learning is easily applicable for other methods since affinity evaluation is only required.

To verify our proposed method one-by-one, we have provided extensive evaluations and ablation studies. Especially, we successfully show that our object constraint learning algorithm enhances tracking speed while maintaining the MOT accuracy. Furthermore, our method achieves the enhanced multi-object tracking performance on the MOT16 benchmark challenge. From the comparison with other state-of-the-art tracking methods, we have verified that our method can achieve a better tracking accuracy and speed. In addition, we expect that our proposed method can be exploited for other multi-object tracking methods, and applied for various fields in real world (e.g., autonomous driving and surveillance system).

For the future work, we focus on improving global models to consider not only the relation between objects but global contexts in spatio-temporal domain. To this end, a transformer model can be adopted since it is effective to learn the global context information. By exploiting the global contextual feature from the transformer, tracking accuracy could be improved further.

## Figures and Tables

**Table 1:** Comparison with recent multi-object tracking methods on the 2016 MOT benchmark challenge. Results are sorted by the setting and MOTA score. More details can be found in the MOT benchmark website (https://motchallenge.net/results/MOT16/, accessed on 28 September 2022). DPM [83] denotes original public detections provided by MOT16. However, detections marked with other names (e.g., FCOS, Faster R-CNN, etc.) mean the refined detections by applying the corresponding detectors.

Method	Setting	Detections	MOTA ↑	IDF1 ↑	MT ↑	ML ↓	FP ↓	FN ↓	ID Sw. ↓	Hz ↑
Tube_TK [82]	Online	FCOS [84]	64.0%	59.4%	33.5%	19.4%	10,962	53,626	1117	1.0
TMOH [61]	Online	Faster R-CNN [62]	63.2%	63.5%	27.0%	31.0%	3122	63,376	635	0.7
MOTRF [81]	Online	YOLOv3 [64]	57.9%	41.7%	28.5%	22.1%	8196	66,538	2051	11.1
LSST16O [13]	Online	DPM [83]	49.2%	56.5%	13.4%	41.4%	7187	84,875	606	2.0
KCF16 [11]	Online	DPM [83]	48.8%	47.2%	15.8%	38.1%	5875	86,567	906	0.1
SOT + MOT [14]	Online	DPM [83]	46.4%	-	18.6%	46.5%	12,491	87,855	404	0.8
DMAN [15]	Online	DPM [83]	46.1%	46.1%	17.4%	42.7%	7909	89,874	532	2.4
oICF [85]	Online	DPM [83]	43.2%	49.3%	11.3%	48.5%	6651	96,515	381	0.4
AM_ADM [32]	Online	DPM [83]	40.1%	43.8%	7.1%	46.2%	8503	99,891	789	5.8
HISP_DAL [19]	Online	DPM [83]	37.4%	30.5%	7.6%	50.9%	3222	108,865	2101	3.3
JCmin_MOT [77]	Online	DPM [83]	36.7%	28.6%	7.5%	54.4%	2936	111,890	667	14.8
GM_PHD_DAL [76]	Online	DPM [83]	35.1%	26.6%	7.0%	51.4%	2350	111,886	4047	3.5
GM_PHD_N1T [78]	Online	DPM [83]	33.3%	22.6%	7.2%	51.4%	1750	116,452	3499	9.9
ApLift [28]	Batch	Faster R-CNN [62]	61.7%	66.1%	34.3%	31.2%	9168	60,180	495	0.6
Lif_T [2]	Batch	Faster R-CNN [62]	61.3%	64.7%	23.2%	34.5%	4844	65,401	389	0.5
TPM [29]	Batch	DPM [83]	51.3%	47.9%	18.7%	40.8%	2701	85,504	569	0.8
MHT_bLSTM [5]	Batch	DPM [83]	42.1%	47.8%	14.9%	44.4%	11,637	93,172	753	1.8
LINF1_16 [6]	Batch	DPM [83]	41.0%	45.7%	11.6%	51.3%	7896	99,224	430	4.2
GMMCP [79]	Batch	DPM [83]	38.1%	35.5%	8.6%	50.9%	6607	105,315	937	0.5
LTTSC-CRF [80]	Batch	DPM [83]	37.6%	42.1%	9.6%	55.2%	11,969	101,343	481	0.6
**MOT_GM (Proposed)**	Online	DPM [83]	43.2%	51.5%	9.0%	54.5%	3481	99,532	484	10.31
**MOT_GM (Proposed)**	Online	CenterNet [86]	64.5%	70.9%	36.4%	20.7%	21,182	42,730	816	6.54

**Table 2:** Comparison with the baseline confidence-based multi-object tracking method on 2016 MOT benchmark train dataset.

Baseline Multi-Object Tracking Method									
**Sequence**	**MOTA ↑**	**MOTP ↑**	**IDF1 ↑**	**MT ↑**	**ML ↓**	**FP ↓**	**FN ↓**	**ID Sw. ↓**	**Hz ↑**
**MOT16-02**	25.76%	75.01%	32.00%	3.70%	53.70%	192	12,982	66	16.33
**MOT16-04**	43.52%	76.89%	40.29%	9.64%	36.14%	1277	25,355	229	12.57
**MOT16-05**	29.41%	74.92%	38.96%	2.40%	51.20%	313	4472	28	21.08
**MOT16-09**	56.91%	73.98%	54.32%	28.00%	8.00%	133	2098	34	16.57
**MOT16-10**	37.24%	73.75%	46.53%	11.11%	48.15%	420	7274	37	16.19
**MOT16-11**	51.32%	78.16%	56.08%	17.39%	50.72%	270	4179	17	16.24
**MOT16-13**	19.15%	71.92%	28.84%	5.61%	66.36%	240	8993	24	17.41
**Total**	37.84%	75.90%	40.89%	8.51%	49.71%	2845	65,353	435	16.02
**Proposed Method**									
**Sequence**	**MOTA ↑**	**MOTP ↑**	**IDF1 ↑**	**MT ↑**	**ML ↓**	**FP ↓**	**FN ↓**	**ID Sw. ↓**	**Hz ↑**
**MOT16-02**	26.31%	75.02%	32.90%	7.41%	55.56%	90	13,001	51	13.11
**MOT16-04**	50.85%	78.05%	61.94%	15.66%	34.94%	165	23,170	38	8.39
**MOT16-05**	28.84%	74.90%	41.33%	1.60%	55.20%	242	4582	28	18.09
**MOT16-09**	57.24%	74.30%	55.01%	20.00%	12.00%	86	2139	23	14.70
**MOT16-10**	38.11%	74.40%	44.91%	12.96%	50.00%	238	7350	34	13.25
**MOT16-11**	51.58%	78.02%	58.52%	14.49%	52.17%	178	4246	18	14.82
**MOT16-13**	17.90%	72.75%	27.63%	3.94%	69.16%	129	9261	10	14.91
**Total**	41.05%	76.69%	51.00%	8.70%	51.84%	1129	63,749	202	12.87

**Table 3:** Comparison with baseline MOT algorithm and MOT with our proposed global models.

Method	Appearance Model	Motion Model	MOTA ↑	MOTP ↑	IDF1 ↑	MT ↑	ML ↓	FP ↓	FN ↓	ID Sw. ↓
M1	Color	Self	37.84%	75.90%	40.89%	8.51%	49.71%	2845	65,353	435
M2	Color	Self, Relation	40.61%	76.54%	48.20%	8.90%	50.68%	1467	63,828	280
M3	Deep	Self, Relation	41.05%	76.69%	51.00%	8.70%	51.84%	1129	63,749	202

**Table 4:** Comparison of appearance models trained with different feature dimensions.

Dimension	MOTA ↑	MOTP ↑	IDF1 ↑	MT ↑	ML ↓	FP ↓	FN ↓	ID Sw. ↓	Hz ↑	MOTA × Hz
**64**	40.50%	76.67%	49.96%	8.32%	52.42%	1273	64,187	234	13.36	541.08
**128**	40.36%	76.69%	49.93%	7.74%	52.80%	1281	64,328	242	13.41	541.28
**256**	39.31%	76.65%	49.08%	6.58%	53.58%	1524	65,160	234	13.34	524.40

**Table 5:** Comparison of multi-object tracking performance with self motion, relation motion, and combined motion models.

Method	MOTA ↑	MOTP ↑	MT ↑	ML ↓	FP ↓	FN ↓	ID Sw. ↓
**Self motion model**	40.81%	76.69%	8.12%	51.64%	1166	63,987	196
**Relation motion model**	40.68%	76.78%	8.70%	52.61%	1126	64,171	201
**Combined motion model**	41.05%	76.69%	8.70%	51.84%	1129	63,749	202

**Table 6:** The MOT performance comparison between multi-object tracking methods with/without the proposed object constraint learning.

MOT without Object Constraint Learning											
**Sequence**	**MOTA ↑**	**MOTP ↑**	**IDF1 ↑**	**MT ↑**	**ML ↓**	**FP ↓**	**FN ↓**	**ID Sw. ↓**	**Appearance** **Updates ↓**	**Motion** **Updates ↓**	**Hz ↑**
**MOT16-02**	26.31%	75.02%	32.90%	7.41%	55.56%	90	13,001	51	600	600	13.11
**MOT16-04**	50.85%	78.05%	61.94%	15.66%	34.94%	165	23,170	38	1050	1050	8.39
**MOT16-05**	28.84%	74.90%	41.33%	1.60%	55.20%	242	4582	28	837	837	18.09
**MOT16-09**	57.24%	74.30%	55.01%	20.00%	12.00%	86	2139	23	525	525	14.70
**MOT16-10**	38.11%	74.40%	44.91%	12.96%	50.00%	239	7350	34	654	654	13.25
**MOT16-11**	51.58%	78.02%	58.52%	14.49%	52.17%	178	4246	18	900	900	14.82
**MOT16-13**	17.90%	72.75%	27.63%	3.74%	69.16%	129	9261	10	729	746	14.91
**Total**	41.05%	76.69%	51.00%	8.70%	51.84%	1129	63,749	202	5295	5295	12.87
**MOT with Object Constraint Learning**											
**Sequence**	**MOTA ↑**	**MOTP ↑**	**IDF1 ↑**	**MT ↑**	**ML ↓**	**FP ↓**	**FN ↓**	**ID Sw. ↓**	**Appearance** **Updates ↓**	**Motion** **Updates ↓**	**Hz ↑**
**MOT16-02**	25.30%	75.44%	31.07%	7.41%	57.41%	111	13,167	44	389	227	13.34
**MOT16-04**	50.53%	78.03%	60.85%	15.66%	34.94%	202	23,280	43	772	318	8.92
**MOT16-05**	26.37%	74.86%	37.41%	0.80%	55.20%	297	4689	34	334	316	18.82
**MOT16-09**	56.13%	74.14%	54.48%	16.00%	12.00%	82	2193	31	193	191	15.12
**MOT16-10**	37.42%	74.32%	46.41%	11.11%	51.85%	237	7425	47	479	241	13.78
**MOT16-11**	50.51%	77.96%	56.68%	11.59%	55.07%	205	4309	26	255	330	15.46
**MOT16-13**	17.65%	72.45%	26.88%	3.73%	70.09%	147	9265	17	433	267	15.44
**Total**	40.36%	76.69%	49.93%	7.74%	52.80%	1281	64,328	242	2855	1890	13.41

## Data Availability

Not applicable.
